# Outcomes of humeral osteotomies versus soft-tissue procedures in secondary surgical procedures for neonatal brachial plexus palsy: a meta-analysis

**DOI:** 10.3389/fsurg.2023.1267064

**Published:** 2023-11-14

**Authors:** Amanda Azer, Dhruv Mendiratta, Anthony Saad, Yajie Duan, Matthew Cedarstrand, Sree Chinta, Aedan Hanna, Dhvani Shihora, Aleksandra McGrath, Alice Chu

**Affiliations:** ^1^ Rutgers New Jersey Medical School, Newark, NJ, United States; ^2^ Department of Statistics, Rutgers University, Newark, NJ, United States; ^3^ Department of Orthopaedics, Rutgers New Jersey Medical School, Newark, NJ, United States; ^4^ Department of Clinical Sciences, Umeå- University, Umeå, Sweden; ^5^ Department of Surgical and Perioperative Sciences, Umeå University, Umeå, Sweden

**Keywords:** neonatal brachial plexus birth palsy, secondary surgery, osteotomy, muscle transfer, tendon transfer

## Abstract

Secondary surgical procedures can be used in brachial plexus birth injury to correct shoulder movement imbalances. This study compares outcomes of the two secondary surgical procedure types: humeral osteotomies and soft tissue procedures. Outcome measures assessed included active and passive internal and external rotation, active and passive abduction and adduction, active and passive flexion and extension, percentage of the humeral head anterior to the middle glenoid fossa, glenoid version, and Mallet Score. Nineteen full-text articles were included in the analysis. Humeral osteotomies resulted in a loss of internal rotation postoperatively (−15.94°). Active internal rotation was not evaluated for soft tissue procedures. All other assessed outcomes were improved postoperatively for bony and soft tissue procedures. Bony procedures exhibited a greater degree of active external rotation postoperatively when compared to soft tissue procedures (+67° vs. +40°). Both bony and soft tissue procedures Improve shoulder function in children with neonatal brachial plexus palsy, however, soft tissue procedures showed greater consistency in outcomes.

**Level of Evidence**: IV

## Introduction

Despite advances in maternal and obstetric care, brachial plexus birth injury (BPBI) remains stable in its prevalence of about 0.174% (1). BPBI is commonly the result of excess traction placed on the head and neck of a baby during birth and is more common in patients weighing greater than 4 kilograms or those with shoulder dystocia (2). Many cases of BPBI resolve spontaneously within the first 6 months of life, however, 10%–30% of deformities persist and require primary and secondary surgeries to regain function lost due to the nerve damage (3). Primary surgeries consist of nerve grafts, transfers, and neurolysis aiming to augment and re-establish a connection to peripheral targets, at a time of profound biological challenges for the developing nervous system (4). However, secondary surgical procedures targeting muscles, joints and bones are necessary if the results of primary nerve surgery or spontaneous recovery were not sufficient or if patients no longer qualify for nerve surgery because of increasing age or complex presenting symptoms (5).

Injured nerve roots can range depending on the extent of injury to the brachial plexus, although the most involved area includes the C5–C7 roots (6), which puts most patients in the class 1 (46%) and 2 (30%) according to Narakas classification (6). This results in effects on muscle groups innervated by the suprascapular nerve and the anterior and posterior divisions of upper trunk-derived nerves, leading to Erb’s Palsy (6). As a result, limited shoulder abduction and an imbalance between internal and external rotation occurs (7). The patients suffer not only from motor deficits limiting use of the upper limb but are affected also by a cosmetic deformity, with issues such as length discrepancy and size of the arm (8). To resolve these deformities a wide range of procedures are available, including those that focus on soft tissue such as muscle transfers and joint releases or those affecting bony elements (9, 10). Bony procedures, such as osteotomies are historically done only by orthopaedic surgeons and involve the usage of hardware, whereas soft tissue procedures range more widely in the type of surgeon completing the procedure and are commonly less invasive than bony procedures. Thus, the knowledge of which procedure is more fitting for what functional deficit is of great value. The literature is also unclear as to which procedure category, soft tissue or bony, produces more consistent results. The aim of this meta-analysis is to compare outcomes of bony versus soft tissue secondary surgical procedures in BPBI patients.

## Materials and methods

### Literature search

**Figure 1:**
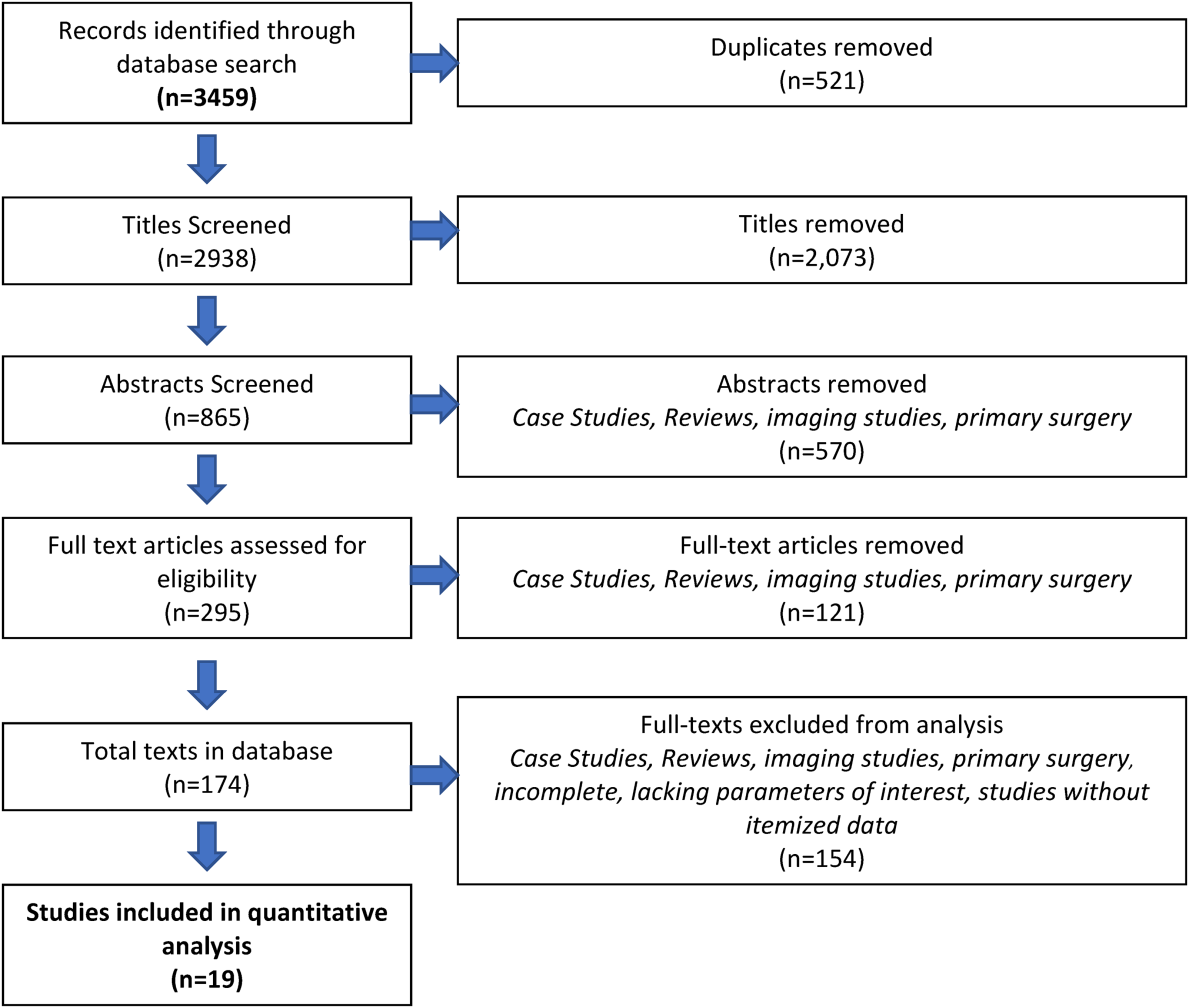
Prisma.

Following PRISMA-IPD guidelines (11), we completed a literature search of the following databases: Cumulative Index to Nursing and Allied Health Literature (CINAHL), PubMed, Scopus, and Web of Science. The search strategy for PubMed is included in Table 1. Articles that met the following inclusion criteria were included in this study. Articles had to be original articles, comprised of randomized control trials, nonrandomized studies, and retrospective case reports/series that described secondary shoulder surgeries on BPBI patients. Patients needed to be less than 18 years of age at the time of surgery, with a minimum of 3 patients in each study. Only papers reporting itemized patient data were included. Three reviewers (AA, AH, DS) screened titles, abstracts, and full-text articles, with each entry being screened by two independent reviewers. Inconsistencies were resolved by two senior authors (AC, AM). The process of inclusion in this study can be found in Figure 1 (PRISMA flow chart).

**Table 1:** All the studies included in the review.

Author	Title	Published year	Number of patients	Age at time of surgery in years	Follow-up time	Category
Lahiji (Lahiji et al., 2017)	Transfer of Pectoralis Major to Subscapularis in the Management of Brachial Plexus Birth Palsy Sequels.	2017	22	4.083333333	4.25	Soft tissue
Pedowitz (Pedowitz et al., 2007)	Arthroscopic treatment of posterior glenohumeral joint subluxation resulting from brachial plexus birth palsy.	2007	22	3.9	3.583333333	Soft tissue
Pöyhiä (Poyhia et al., 2011)	Treatment of shoulder sequelae in brachial plexus birth injury.	2011	31	6.42	3.8	Soft tissue
Abid (Abid et al., 2012)	Arthroscopic release for shoulder internal rotation contracture secondary to brachial plexus birth palsy: clinical and magnetic resonance imaging results on glenohumeral dysplasia.	2012	6	1.9	5	Soft tissue
Breton (9)	Arthroscopic release of shoulder contracture secondary to obstetric brachial plexus palsy: retrospective study of 18 children with an average follow-up of 4.5 years.	2012	18	4.17	4.5	Soft tissue
Armangil (Mehmet Armangil, 2012)	Arthroscopic release of the subscapularis for shoulder contracture of obstetric palsy	2012	6	5.1	2.79	Soft tissue
Andres-Cano (Andres-Cano et al., 2015)	Arthroscopic treatment for internal contracture of the shoulder secondary to brachial plexus birth palsy: report of a case series and review of the literature.	2015	5	2.8	1.66	Soft tissue
Mehlman (Mehlman et al., 2011)	Arthroscopically assisted Sever-L'Episcopo procedure improves clinical and radiographic outcomes in neonatal brachial plexus palsy patients.	2011	50	5.1	2.5	Soft tissue
Waters (12)	Effect of tendon transfers and extra-articular sof-tissue balancing on glenohumeral development	2005	25	3.5	3.58	Soft tissue
Al-Qattan (Al-Qattan and Al-Kharfy, 2015)	External Rotation Osteotomy of the Humerus to Salvage the Failed Latissimus Dorsi Transfer in Children With Erb Birth Palsy and Supple Congruent Shoulders.	2015	6	2	2	Bony
Kirkos (Kirkos and Papadopoulos, 1998)	Late treatment of brachial plexus palsy secondary to birth injuries: rotational osteotomy of the proximal part of the humerus.	1998	22	10.25	14	Bony
Bertelli (Bertelli, 2009)	Lengthening of subscapularis and transfer of the lower trapezius in the correction of recurrent internal rotation contracture following obstetric brachial plexus palsy	2009	7	7	4	Soft tissue
Goddard (Goddard and Fixsen, 1984)	Rotation osteotomy of the humerus for birth injuries of the brachial plexus.	1984	10	7.67	5.5	Bony
Dedini	Comparison of pediatric outcomes data collection instrument scores and range of motion before and after shoulder tendon transfers for children with brachial plexus birth palsy.	2008	234	6.310.15	0.6875	Soft tissue
Chomiak (Chomiak et al., 2014)	Muscle transfers in children and adults improve external rotation in cases of obstetrical brachial plexus paralysis: a comparative study.	2014	15	9.33	1	Soft tissue
Maurya (Maurya et al., 2021)	Conjoint muscle transfer and subscapularis slide in brachial plexus birth palsy: Clinical outcomes in shoulder functions.	2021	18	4.64	7.9666666	Soft tissue
VanHeest (13)	Glenohumeral dysplasia changes after tendon transfer surgery in children with birth brachial plexus injuries.	2010	26	3.66666666	1.5	Soft tissue
Ghaly (Ghaly et al., 2017)	Remodeling after arthroscopic reduction of glenohumeral joint in adduction internal rotation shoulder deformity in obstetric brachial plexus palsy	2017	21	2.16666666	1.42	Soft tissue
Al-Qattan (Al-Qattan et al., 2009)	Long-term results of low rotation humeral osteotomy in children with Erb’s obstetric brachial plexus palsy.	2009	17	6	10	Bony

### Data extraction and screening

Data extracted from each article included the year of publication, the number of patients included in the study, the age, and sex of the patients, follow-up time after the procedure, and all outcome measures. Outcome measures included active and passive internal and external rotation, active and passive abduction and adduction, active and passive flexion and extension, percentage of the humeral head anterior to the middle glenoid fossa (PHHA), glenoid version, overall Mallet Score and commonly reported Mallet Score subcategories such as abduction, hand to mouth, hand to neck, external rotation, and hand to spine. Hand to belly scores were not provided by many of the papers analyzed, thus were not included in the overall analysis. The procedures described in the included studies were classified as bony and soft tissue procedures. Bony procedures were comprised of studies that assessed humeral osteotomies and soft tissue category included studies describing muscle and tendon transfers, releases, and transpositions and arthroscopic joint releases.

### Statistical analysis

Paired *t*-tests and paired Wilcoxon tests were completed for analysis within each group (bony and soft tissue procedures) for pre-and post-operative outcome measures. Unpaired *t*-tests and unpaired Wilcoxon tests were completed to compare data points between groups’ outcome measures. Wilcoxon tests took precedence in the groups with smaller data sets or those where the data did not present a normal distribution. To assess variance, empirical distributions of the data from groups were calculated and compared using the two-sample Kolmogorov-Smirnov (KS) test. The two-sample KS test is a statistical test that is being used here to specifically compare the empirical distributions of outcomes pre- and post-operatively to see if there is any statistically significant difference between distributions suggesting differences in consistency of the results.

## Results

### Review characteristics

**Figure 2:**
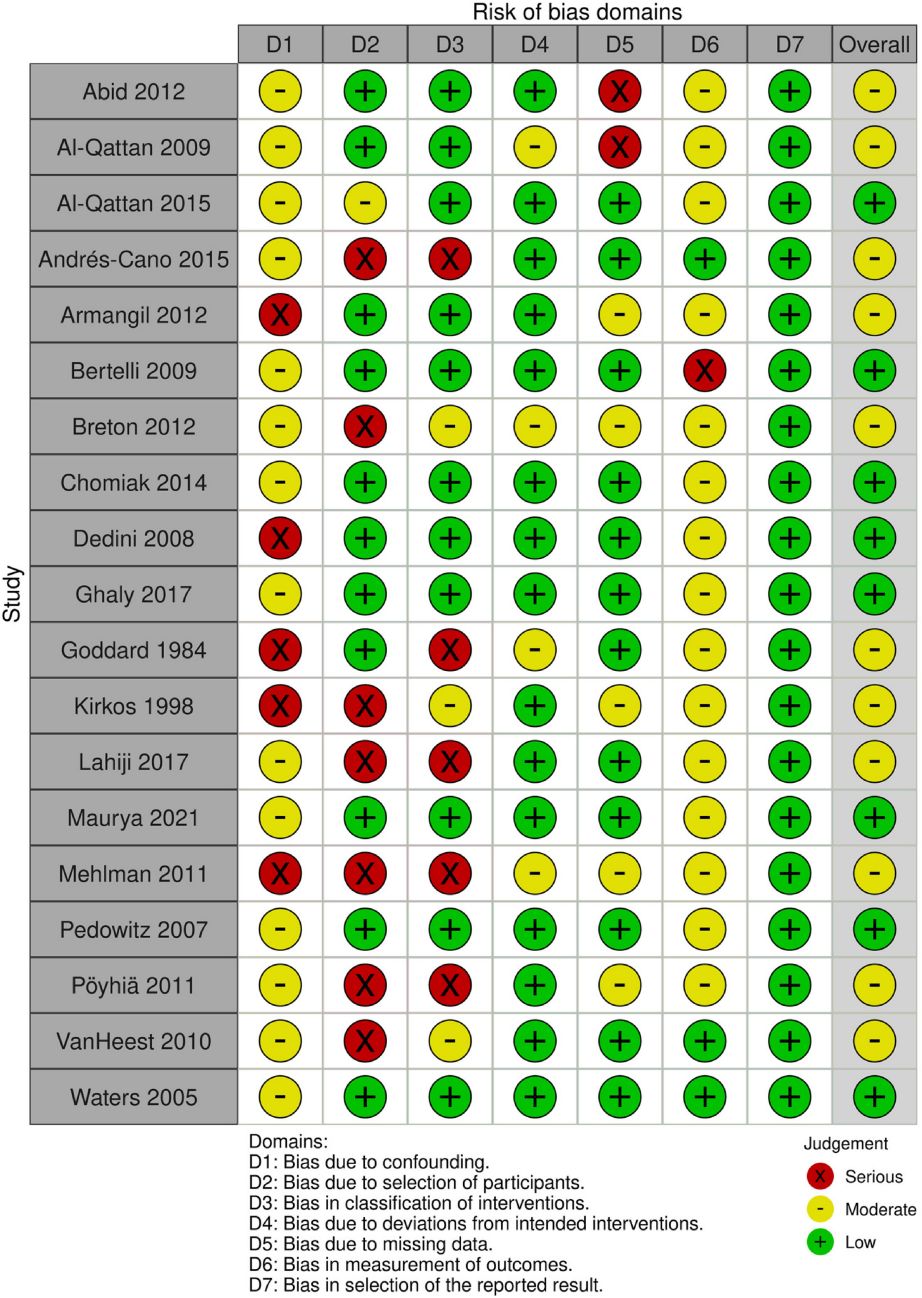
Irobins.

Database searches resulted in a total of 3,459 articles; of those, 2,938 titles were screened after removing duplicates. Three independent reviewers assessed 865 abstracts and 295 full-text articles. After the removal of full-text articles that did not have itemized patient data, 19 full-text articles were included in this study. More information regarding the process can be found in the PRISMA flowchart (14) (Figure 1). All included studies and their characteristics are listed in Table 1. Further information regarding the risk assessment can be found in Figure 2.

### Risk of bias and quality of evidence

To assess the bias assessment, the iROBINS tool was used. Of the 19 articles, 8 articles were found to have overall low bias, and 11 articles were found to have moderate bias. All articles were found to have low bias in the selection of reported results, and 15 articles were found to have low bias due to deviations from intended interventions while the other 4 had moderate bias. The category that had the highest number of articles with bias was bias due to selection of participants, in which 7 articles were found to have high bias.

### Outcomes and interventions

Nineteen studies presented itemized data on 347 patients. Bony procedures had an average of 14.6 (SD 6.5) patients in each study while soft tissue procedures had an average of 18.3 patients (SD 11.5). 14 studies were classified as soft tissue and included 274 patients, and 4 were classified as bony and included 73 patients. One study analyzed both bony and soft tissue procedures, thus the patients were divided into each of their respective sections. Age at the time of surgery was analyzed, and found to have no significant difference between that of bony and soft tissue procedures. From these data, we have compared bony and soft tissue procedures and within each of those groups analyzed differences between pre-and post-operative values. Outcomes that were compared were those that were presented by more than one article. This included the following outcomes: Mallet Score and its subcategories (hand-to-mouth, hand-to-neck, hand-to-spine, external rotation, and abduction), active external rotation, active abduction, active internal rotation, percentage of the humeral head anterior to the middle glenoid fossa (PHHA) and glenoid version.

**Figure 3:**
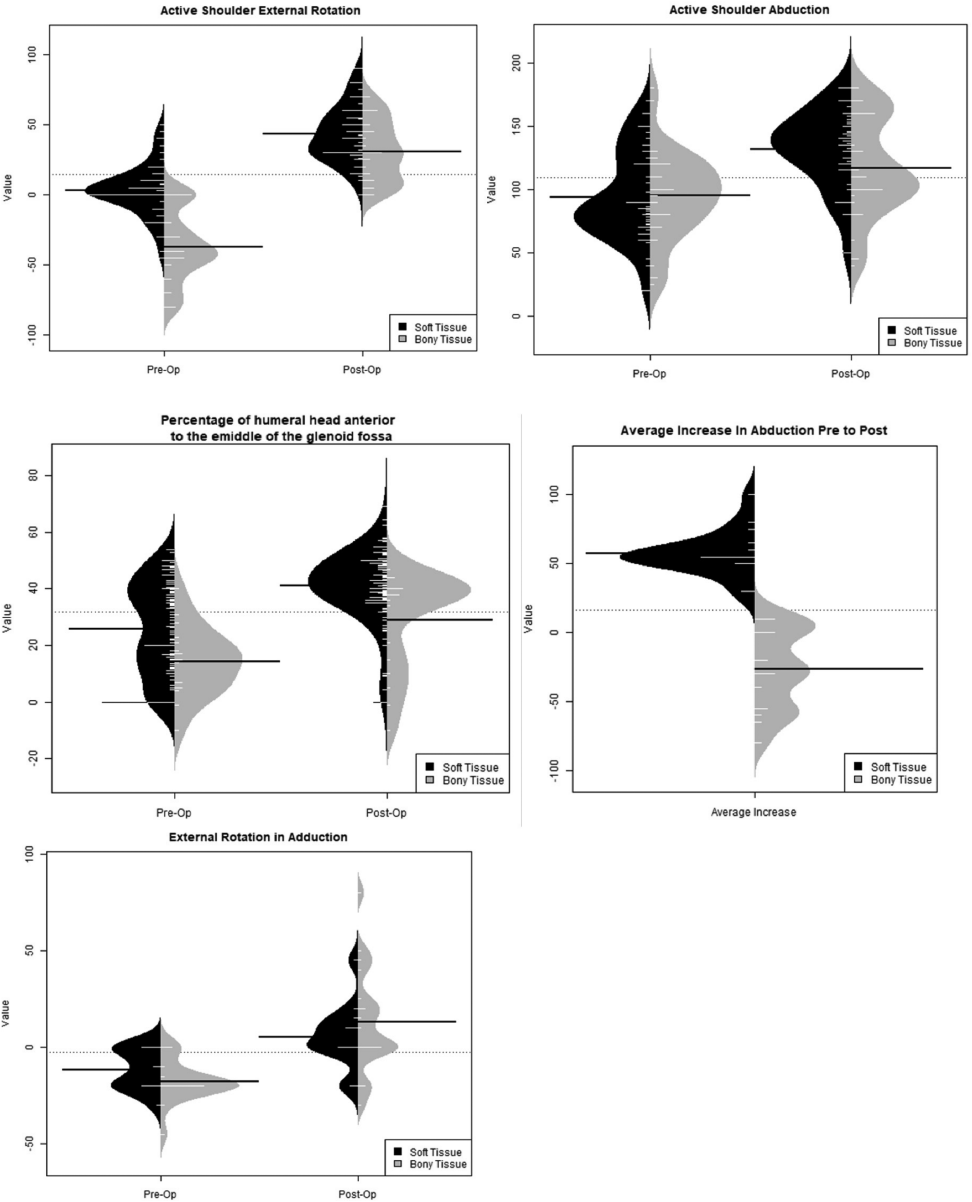
Ks test for bony versus soft tissue distributions.

**Figure 4:**
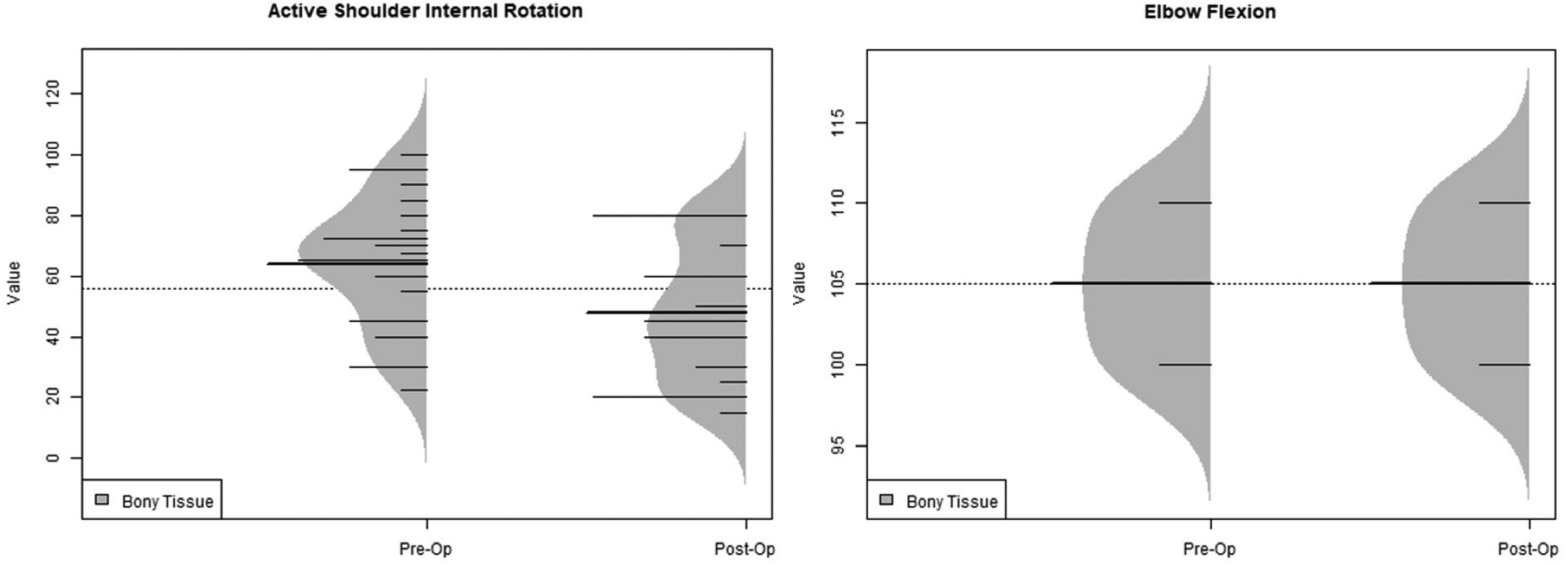
Ks test for bony distributions.

**Figure 5:**
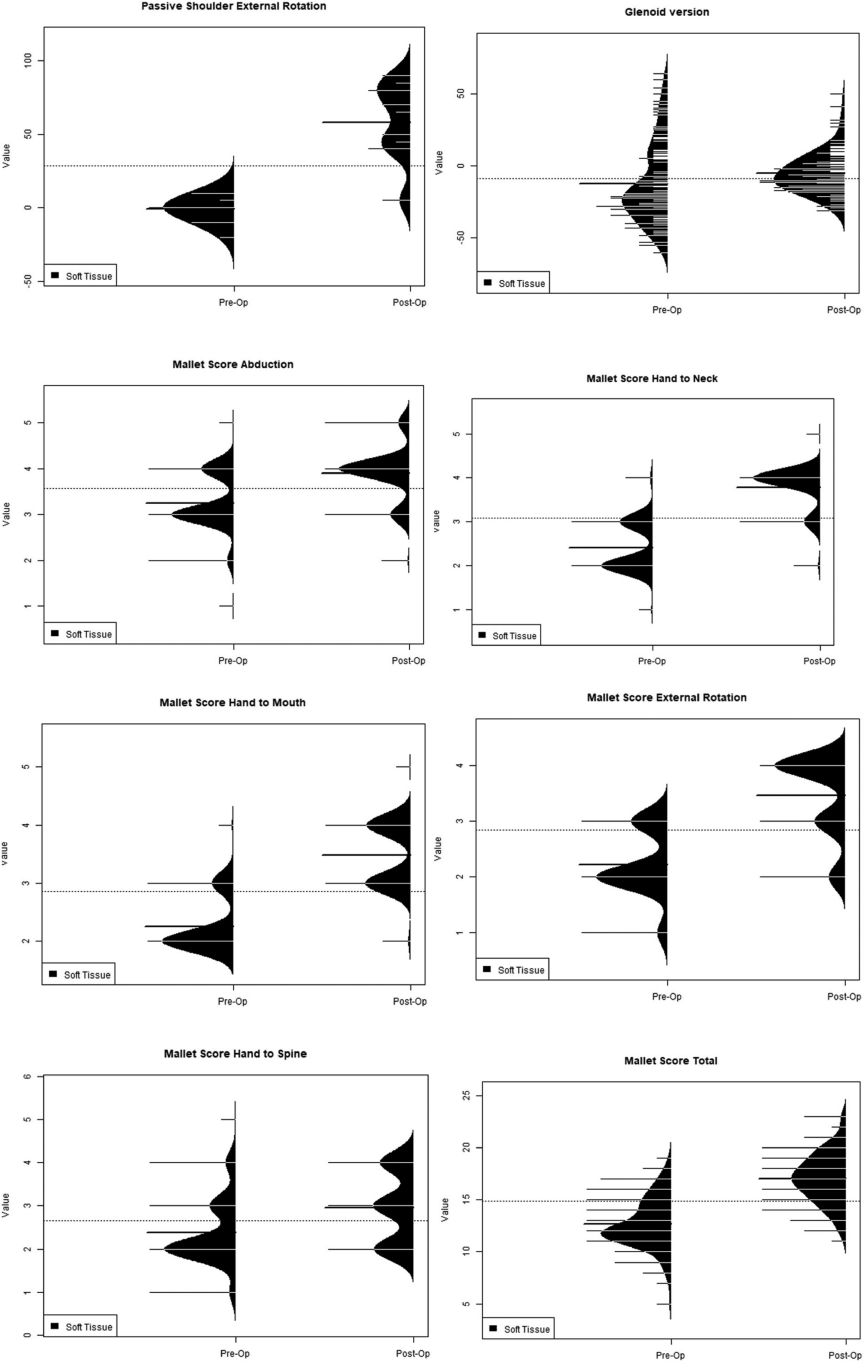
Ks test for soft tissue distributions.

Bony procedures were found to have statistically higher post-operative values when compared to pre-operative values for active external rotation, active abduction, PHHA, and total Mallet Score. Pre-operative active internal rotation (63.75 vs. 47.81) was found to be significantly greater, indicating a loss of internal rotation postoperatively (Table 2). Soft tissue procedures were found to have statistically greater post-operative values for active external rotation, active abduction, PHHA, glenoid version, and Mallet Score abduction, hand-to-neck, hand-to-mouth, external rotation, and hand-to-spine when compared to preoperative values (Table 3). Active internal rotation data was not available for analysis within the soft tissue group. An analysis using the KS test to asses the distributions of these range of motion variables also yielded the same significant differences (Figures 3–5).

**Table 2:** Variables that were significantly different for bony versus soft tissue pre-and post-operative outcomes.

Subgroup	Outcome measure	Soft tissue mean (SD)	Number of patients soft tissue	Bony mean (SD)	Number of patients bony	KS test
Pre-Operative	Active External Rotation	3.07 (17.95)	67	−37.03 (24.90)	32	7e-9
	PHHA	25.76 (16.56)	121	14.22 (12.10)	18	0.006814
Post-Operative	Active External Rotation	43.22 (20.12)	67	30.78 (21.82)	32	0.03422
	Active Abduction	131.45 (30.61)	51	116.84 (38.79)	38	0.00394
	PHHA	40.98 (12.48)	121	28.83 (17.48)	18	0.004915

**Table 3:** Pre- and post-operative outcomes comparison for bony and soft tissue procedures.

Subgroup	Outcome measure	Pre-Op Mean (SD)	Post-op mean (SD)	Paired *t*-test/paired Wilcoxon test *p*-value	Delta
Bony	Active External Rotation	−37.03 (24.90)	30.78 (21.82)	2.2e-16	67.81
	Active Internal Rotation	63.75 (20.94)	47.81 (22.36)	0.002278	15.94
	Active Abduction	95.13 (34.06)	116.84 (38.79)	9.342e-9	21.71
	PHHA	14.22 (12.10)	28.83 (17.48)	0.000338	14.61
Soft Tissue	Active External Rotation	3.07 (17.95)	43.22 (20.12)	2.2e-16	40.15
	Active Abduction	94.04 (35.39)	131.45 (30.61)	2.329e-13	37.41
	PHHA	25.76 (16.56)	40.98 (12.48)	2.2e-16	15.22
	Glenoid Version	−12.14 (28.19)	−5.01 (14.40)	0.0001363	7.13
	Mallet Score Abduction	3.25 (0.61)	3.90 (0.58)	4e-15	0.65
	Mallet Score Hand to Neck	2.41 (0.57)	3.78 (0.50)	1e-14	1.36
	Mallet Score Hand to Mouth	2.25 (0.46)	3.48 (0.57)	5e-15	1.23
	Mallet Score External Rotation	2.22 (0.59)	3.46 (0.73)	2.2e-16	1.24
	Mallet Score Hand to Spine	2.37 (0.75)	2.94 (0.80)	2e-9	0.57
	Total Mallet Score	12.65 (2.40)	17.04 (2.44)	2e-9	4.39

**Figure 6:**
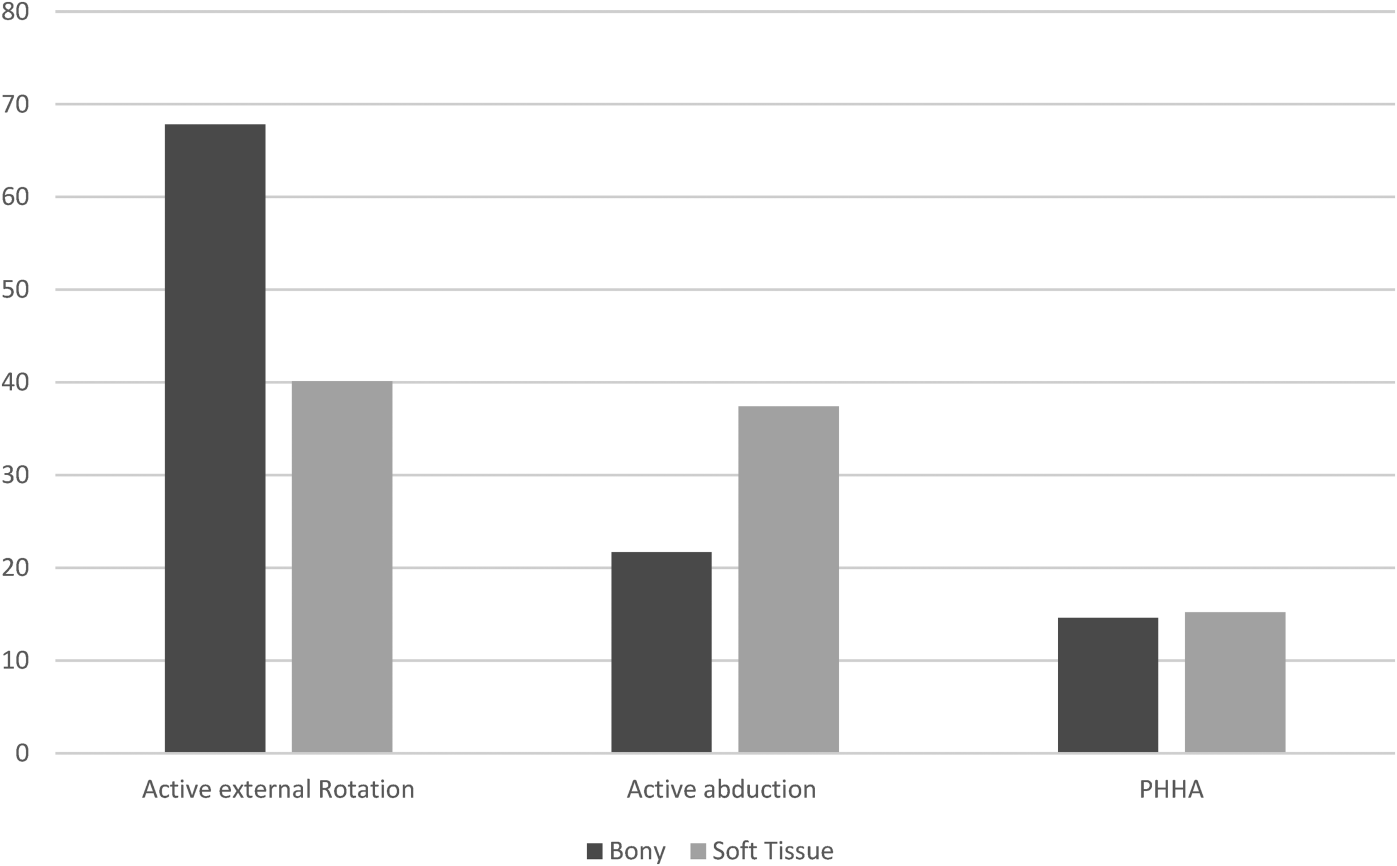
Itemized data change in degrees in active motion from pre- to post-operative.

When comparing pre-and post-operative changes between the groups, bony procedures were found to have greater increases for external rotation when compared to soft tissue procedures. Bony procedures had a mean of 67-degree increase in active external rotation post-operatively while soft tissue procedures only had a 40-degree increase (Figure 6). This indicates higher gains of external rotation arc for patients undergoing bony procedures even if their preoperative external rotation was poorer than in patients undergoing soft tissue procedures. Other variables such as PHHA and active abduction showed similar increases between both groups, with soft tissue procedures having significantly higher pre- and post-operative values and greater overall increases compared to bony procedures (Figure 6).

The Kolmogorov-Smirnov (KS) test was also used to compare the shapes of distributions (Figure 3). PHHA distributions narrowed post-operatively for soft tissue procedures and remained similar for bony procedures. Distributions for bony procedures widened for active shoulder abduction but were narrowed for soft tissue procedures. Regarding active external rotation, bony distributions narrowed while soft tissue procedures widened postoperatively. Bony procedures showed a similar distribution for active internal rotation pre-and post-operatively, although the distribution shifted to lower values overall (Figure 4). Soft tissue procedures had a narrowed distribution post-operatively for glenoid version (Figure 5), which was improved. Mallet score subcategories, including external rotation, hand to mouth, hand to neck, abduction, and hand to spine all had similar distributions post-operatively, though the peaks of each distribution shifted to higher values for soft tissue procedures. Changes in distributions were all found to be statistically significant when comparing pre- and post-operative values and those comparing bony versus soft tissue (Figures 3–5).

## Discussion

Overall, our analysis showed that soft tissue procedures show significant increases in more range of motion variables than bony procedures do. No significant difference was found pre-operatively for active abduction, but post-operatively soft tissue procedures were found to have higher values than bony procedures, showing that soft tissue procedures are better at addressing that deficit. Distribution analysis showed that soft tissue procedures had more narrowed variances post-operatively, while bony procedures had some narrowed and some widened distributions. This indicates that soft tissue procedures have more consistency in their post-operative results for almost all range of motion variables.

The extent to which variables increased differed between both procedure types. Bony procedures had a much greater increase in active external rotation post-operatively when compared to soft tissue procedures. Our results also indicated that soft tissue procedures had higher pre-operative degrees of external rotation (higher starting point), as presumably patients with higher degrees of shoulder internal rotation contracture were being offered bony procedures. This suggests that, although soft tissue procedures yielded a greater overall external rotation value post-operatively, this was secondary to the fact that the patients selected for the bony procedures are those who are initially much worse when external rotation is considered.

Given the indication of osteotomies to be used in those with significant internal rotation contracture or advanced glenohumeral deformity, it is no surprise that measures of glenohumeral deformity vary between the two groups (12). Therefore, measures of preoperative dysplasia, including active abduction and PHHA should vary between the groups, as the selection of patients planning to undergo the procedures vary in functional and aesthetic quality. Further, we would expect that glenoid version and Mallet scores would reflect this, as well, although there was limited data on these measures in the bony group. With respect to active abduction and PHHA post-operatively, soft tissue procedures not only resulted in greater values but also showed greater increases, when compared to bony procedures. The reasoning for this finding can be attributed to osteotomies not addressing the underlying structural and functional abnormalities and instead aiming for a cosmetic improvement in shoulder subluxation (15). However, osteotomies of the humerus are not designed with the aim of improving abduction, thus, soft tissue procedures resulting in greater gains in external rotation and abduction is not surprising. Nonetheless, our findings support the assertion that soft tissue procedures have a well-documented history of improving glenohumeral dysplasia (13). Of course, timely intervention is a necessary step in ensuring these functional improvements.

An analysis of distributions was completed, using the Kolmogorov-Smirnov (KS) test, to describe the reproducibility and consistency of outcomes for bony and soft tissue procedures. Soft tissue procedures had reliably achieved active abduction and glenoid version, with a narrow distribution of the results, while other parameters, such as external rotation presented a wider distribution of results, leading to less reliable outcomes. We have observed that bony procedures improve reliably active external rotation, as evidenced by narrow distribution**,** but are less reliable for other range of motion outcomes, such as active abduction, which presented with wider distribution postoperatively. Both bony and soft tissue procedures affect the remodeling of the glenohumeral joint positively and have consistency in these results as shown in improved PHHA and narrowed distribution.

The finding that osteotomies resulted in decreased internal rotation is a planned outcome, as the purpose remains to place the limb in a better functional and aesthetic position (16). Thus, the finding of a decrease in internal rotation is expected with an increase in external rotation, as evidenced by our study. However, a commonly reported complication in soft tissue procedures is loss of midline function, often requiring rebalancing procedures (17). Our lack of evidence regarding active internal rotation pre- and post-operatively in the soft tissue procedure articles we assessed requires further contextualization by the literature. Internal rotation deficits have been reported regularly after these release and relocation procedures (18). As much as a 42-degree reduction has been noted after arthroscopic tenotomy of the subscapularis tendon, often requiring secondary internal rotation osteotomies for correction (19). Recognition of such outcomes in soft tissue procedures has prompted the modification of these soft tissue techniques and the addition of a sixth Mallet Score: hand-to-belly (20). Unfortunately, the articles selected for this study rarely reported the outcome measure due to its relative novelty. Loss of midline function following soft tissue procedures requires a more thorough review and scrutiny. During the consent process patients and caregivers expect information on likely complications as this can help patients to accept the unwanted outcomes when complications occur (21).

Age may play a role in the treatment of pediatric patients with deformities secondary to NBPP. Although we did not find a significant difference between the age of patients undergoing bony versus soft tissue procedures, the effects of growth on the long-term outcomes of pediatric orthopaedic NBPP procedures have not been studied. A study reviewing derotational osteotomies in cerebral palsy patients found that the recurrence rate of femoral anteversion and gait abnormalities in this population was 9%, with age at the time of surgery playing a role (22). Conversely, if the osteotomy is too dramatic, growth may lead to the opposite deformity of excess loss of internal rotation taking place in children after growth. With regards to soft tissue procedures, in children who have already achieved skeletal maturity, they may not be as useful to restore motion in these patients. Thus, more research is warranted to understand the effects of age and growth on these outcomes.

Overall, the outcomes of these studies showed changes post-operatively and over the course of the follow-up period presented. However, due to these patients not being followed during or after puberty, between the ages of 8–14, it is unclear whether these gains in motion are maintained after that. This indicates the need for further studies to address this and understand the longevity of the gains found and whether there is loss after puberty.

This study is limited in that it includes only 19 studies, of which at most, 8 of the studies collected the same outcome. This discrepancy results from a lack of uniformity in which variables to collect and makes a comparison between the types of procedures completed, their efficacy, and success very challenging. A worldwide iPluto study aimed at finding a consensus regarding what outcomes were best fit to review BPBI procedures and outcomes. This study found that follow-up and re-evaluation of post-operative outcomes should be completed at 1/3/5/7 years and outcomes that should be collected include passive and active motion including shoulder internal and external rotation, elbow and wrist flexion and extension, pronation and supination and Mallet Score. These outcomes with respect to shoulder function include active external rotation, active abduction, and Mallet Score and its subcategories. This indicates a need for more uniform data-collecting measures, in line with the findings of iPluto study, to allow for better use of published literature (3).

## Conclusion

Both bony and soft tissue procedures have the ability to provide gains in various ranges of motion variables. Bony procedures provide a greater increase in active external rotation; however, soft tissue procedures provide greater increases across all other variables including active abduction, PHHA, and Mallet Score subcategories. Soft tissue procedures also have more consistency in their post-operative results, as shown by their more narrowed distributions.

## Data Availability

The original contributions presented in the study are included in the article/Supplementary Material, further inquiries can be directed to the corresponding author.
